# Genetic Diversity and Population Parameters of Sea Otters, *Enhydra lutris*, before Fur Trade Extirpation from 1741–1911

**DOI:** 10.1371/journal.pone.0032205

**Published:** 2012-03-05

**Authors:** Shawn Larson, Ron Jameson, Michael Etnier, Terry Jones, Roberta Hall

**Affiliations:** 1 Research/Animal Health, Seattle Aquarium, Seattle, Washington, United States of America; 2 Western Ecological Research Center, United States Geologic Survey (Retired), Philomath, Oregon, United States of America; 3 Department of Anthropology, University of Washington, Seattle, Washington, United States of America; 4 Social Sciences Department, California Polytechnic State University, San Luis Obispo, California, United States of America; 5 Department of Anthropology, Oregon State University, Corvallis, Oregon, United States of America; Barnard College, Columbia University, United States of America

## Abstract

All existing sea otter, *Enhydra lutris*, populations have suffered at least one historic population bottleneck stemming from the fur trade extirpations of the eighteenth and nineteenth centuries. We examined genetic variation, gene flow, and population structure at five microsatellite loci in samples from five pre-fur trade populations throughout the sea otter's historical range: California, Oregon, Washington, Alaska, and Russia. We then compared those values to genetic diversity and population structure found within five modern sea otter populations throughout their current range: California, Prince William Sound, Amchitka Island, Southeast Alaska and Washington. We found twice the genetic diversity in the pre-fur trade populations when compared to modern sea otters, a level of diversity that was similar to levels that are found in other mammal populations that have not experienced population bottlenecks. Even with the significant loss in genetic diversity modern sea otters have retained historical structure. There was greater gene flow before extirpation than that found among modern sea otter populations but the difference was not statistically significant. The most dramatic effect of pre fur trade population extirpation was the loss of genetic diversity. For long term conservation of these populations increasing gene flow and the maintenance of remnant genetic diversity should be encouraged.

## Introduction

The sea otter, *Enhydra lutris*, is a lutrine carnivore in the mustelid family, and the only member of the genus *Enhydra*. The species is represented by three subspecies defined by skull morphometrics: the Russian, *E.l. lutris*; the Northern, *E.l. kenyoni*; and the Southern, *E.l. nereis* [1]. They are thought to have evolved exclusively in the North Pacific from a middle Pliocene ancestor, *Enhydritherium* [2], [3]. Sea otters are amphibious but almost entirely aquatic, with all life cycle activities occurring in the water. However they have limited aquatic adaptations such as relatively shallow diving and relatively short breath holding capacity that keeps them in the shallower, near shore environments (generally less than 30 m) [3].

Sea otters are sexually dimorphic with males typically 34% heavier and 8% longer than females [3]. Male sea otters weigh 30–45 kg and are between 129–150 cm long while females weigh 20–30 kg and are between 119–140 cm, with the Northern sea otter larger than the Southern [1], [3]. They have the thickest fur in the animal kingdom, with some estimates of up to over 1,000,000 hairs per square inch within the densest areas of the pelt [3], [4]. This extremely dense fur traps a layer of air next to the skin, thereby creating an insulating barrier to the cold waters of the North Pacific. This dense fur and air combined with the sea otter's specialized oil glands enhance the water repellent quality of the fur and the ability to keep their skin warm and dry [3].

Sea otter populations have suffered from historical periods of population fragmentation due to extirpations associated with significant human hunting for their luxurious pelts. Sea otters once ranged throughout coastal regions of the north Pacific rim from the islands of northern Japan to central Baja California, Mexico [4]. They were hunted to near extinction throughout this range resulting in a loss of 99% of their original numbers during the fur trade of the 18th and 19th centuries, beginning in 1741 and ending in 1911 when they received protection under the International Fur Seal Treaty [4]. After the fur trade extirpation, only 1% of the estimated original sea otter population remained in approximately 11 geographically isolated populations [4]. Those formed the remnant populations in California, south-central Alaska, the Aleutian, Commander and Kuril Islands, and the Kamchatka Peninsula. By the 1970s, a few sea otter populations had recovered to pre-exploitation levels, but the majority of historic sea otter habitat remained vacant along the west coast of North America from Prince William Sound, Alaska, southward to California [3], [4], [5].

In an effort to re-establish sea otter populations throughout their former range, management authorities made several translocations from the 1950s through the 1970s [6]. In total, 715 otters were captured at Amchitka Island in the Aleutian chain and Prince William Sound, Alaska, and then released at various unoccupied habitats in Alaska, British Columbia, Washington and Oregon. Release locations included the Pribilof Islands in western Alaska, several locations in southeast Alaska, the Bunsby Islands in British Columbia, Pt. Grenville and La Push in Washington, and Port Orford and Cape Arago in Oregon [6]. The translocations to Washington, Oregon and the Pribilof Islands included only animals captured at Amchitka, and only the Washington effort was successful [6], [7]. The translocations to Southeast Alaska and British Columbia (off the west coast of Vancouver Island) included a mix of Amchitka and Prince William Sound animals, and both were successful [6], [7]. Translocation distances varied from approximately 750 km between Amchitka and the Pribilofs to over 5000 km between Amchitka and Oregon. In spite of these successful translocation efforts, sea otter populations today remain fragmented, with many extant populations geographically separated resulting in the cessation of gene flow among groups [7].

Knowledge of the geographic location of surviving sea otter populations during periods of population bottlenecks and of their persistence is limited, although some post-bottleneck survey data are available for estimating population growth rates [7]. Assumed minimum population sizes of the remnant sea otter populations range from 10 to 40 animals and estimated bottleneck durations range from eight to 44 years [7]. These small surviving populations were and still are separated by several hundred km (Commander and Aleutian Islands) to several thousand km (Alaska and California). This geographic separation has essentially created a barrier to gene flow between surviving populations as sea otters are thought to be capable of moving no more than a few hundred km in a single generation [8]. This isolation of the remnant populations from each other, caused by the fur trade extirpation, is thought to have influenced and changed the historical genetic relationships among populations, through many factors such as loss of gene flow between adjacent groups, small founder population sizes, fixation of alleles and genetic drift.

Given this history of population extirpation and fragmentation, it is evident that all extant sea otter populations incurred population bottlenecks of varying severity and duration. The impact of these bottlenecks on genetic variation and genetic relationships (genetic population structure) within surviving sea otter populations remains unclear, inspiring several studies [7], [9], [10], [11], [12], [13]. For example, restriction fragment-length polymorphism (RFLP) and nucleotide sequence analysis of the mtDNA D loop control region analysis of mitochondrial DNA (mtDNA) was used to compare northern and southern sea otters [9], [13], [14]. The haplotype distributions observed in these studies support geographically distinct haplotypes between the two populations but with relatively small genetic distances differences between northern and southern sea otters. There was also relatively low genetic variation within the populations (mean nucleotide sequence divergence of 0.3% for California, 0.6% for Alaska, and 0.3–0.6% between populations) [14].

A survey of genetic variation in several extant sea otter populations has revealed levels of variation in nuclear microsatellites that are also relatively low in both remnant and translocated populations, and comparable to variation in several other mammalian species that have experienced severe population bottlenecks [7], [11], [15]. Previous data from one extinct pre-fur trade sea otter population from Washington revealed that sea otters have lost half of their historical genetic diversity due to fur trade extirpation [12]. However, it is still unknown if this high diversity was found in all pre-extirpation populations. Aguilar et al. 2008 suggest that the low diversity in the current California population is an artifact of an older bottleneck pre-dating the fur trade of the 18^th^ and 19^th^ centuries [15]. Thus the pattern of genetic diversity and also the extent of gene flow or genetic relationships between pre-fur trade sea otter populations remain unknown. In addition it is not clear whether the population differences seen in modern sea otter populations are a consequence of the fur trade or an earlier bottleneck.

To test the hypothesis that all modern sea otter populations have potentially altered inter-population genetic relationships due to fur trade extirpation and population fragmentation we obtained genotypes for five microsatellite loci from DNA extracted from bones of sea otters throughout the range that lived prior and up to the fur trade. We then compared genetic diversity and population structure from these extinct populations to similar data previously reported for extant sea otters [11]

## Materials and Methods

Five microsatellite loci (Mvi 57 and Mvi 87 [16], Mvis 72 and Mvis 75 [17], and Lut 453 [18]) were collected sea otter samples from five pre-fur trade populations and compared to the same loci found in samples from five modern sea otter populations of approximately the same sample size. The resulting five populations were included in the analysis because the population sample sizes were large enough to compare with extant data sets. Final sample sizes for the five pre-fur trade populations from south to north were: California (OLDCA, N = 98), Oregon (OLDOR, N = 40), Washington (OLDWA, N = 34), Alaska (OLDAK, N = 56), and Russia (OLDRU, N = 39). The five extant sea otter populations from south to north were: California (CA, N = 63), Washington (WA, N = 33), South East Alaska (SEAK, N = 25), Prince William Sound (PWS, N = 35), and Amchitka Island (AM, N = 40). The modern populations comprise two recognized subspecies *E. l. kenyoni* (AM, SEAK, PWS, and WA) and *E. l. nereis* (CA) [1].

Genetic data from modern populations and all genetic methods for modern sea otters are described in detail in Larson et al. 2002a [11]. We used both flipper tissue and whole blood samples for DNA extraction. Flipper plugs were preserved in 100% ethanol or frozen at −20°C or −40°C until analysis. Whole blood samples were spun to obtain serum or plasma soon after collection, and then frozen at −20°C until analysis. Alternatively, we preserved an aliquot of whole blood in EDTA, and samples were stored at −20°C or −40°C prior to DNA isolation. DNA was extracted from tissue using a standard phenol–chloroform method [19], resuspended in 100 µl of Tris Ethylenediaminetetraacetic acid (Tris-EDTA) buffer and then stored at −20°C or −80°C for ≤1 yr. DNA from whole blood was extracted using the QIAamp Blood and Tissue Kit (Qiagen, Valencia, CA, USA).

The pre-fur trade samples were taken from curated archaeological museum specimens of sea otter bones recovered from aboriginal midden deposits. The ages of the samples varied but were between 3000-100 years before present (YBP).

All of the 98 OLDCA sea otter remains were obtained from the Diablo Canyon archaeological site (CA-SLO-2) on the central coast of California in San Luis Obispo County. The site was excavated in the 1960s and was originally reported by Greenwood in 1972 [20]. With a basal occupation dated to ca.10,000 YBP, it is one of the oldest known sites on the mainland California coast [21]. The otter remains were recovered only from the upper two thirds of the deposit, and the oldest specimens (N = 5) date to ca 3000 YBP. The remainder date to the late Holocene, ca. 3000-200 YBP and represent animals that are ancestral to the sea otter population living in California today.

The 40 OLDOR samples were from five archaeological sites in Oregon: near Little Whale Cove (35-LNC-43) on the northern Oregon coast [22], near the mouth of the Umpqua River (35-DO-83) [22], near Seal Rock State Park (35-LNC-14) on the central Oregon Coast [22], and two sites (OR-CS-43 and OR-CS-3) near the mouth of the Coquille River [23]. All specimens were curated at the Department of Anthropology at Oregon State University and were from levels dated to between 300 and 3000 YBP. The sea otter is currently extinct in Oregon.

The 34 OLDWA genetic samples were obtained from archaeological bone samples from the Makah Indian village site of Ozette, near Neah Bay, WA. Excavated materials are currently curated by the Makah Cultural and Resource Center in Neah Bay. Although the Ozette village appears to have been occupied for approximately 2000 years [24], stratigraphic evidence indicates that the sampled bones represent sea otters that lived during the interval from 450 to 100 YBP [25], [26]. The pre-fur trade remains were from an extinct Washington sea otter population and are not related to the contemporary WA sea otters, all of which derive from translocated Alaskan otters [6].

OLDAK comprised samples from several different sites. The majority of the 56 OLDAK samples (42) were obtained from skulls taken during the fur trade and curated at the Smithsonian Institution in Washington DC. They range from 100–200 YBP and were taken from various locations throughout the Aleutian Islands to Prince William Sound. They are ancestral to the current Alaskan sea otter population. Eight samples were from the Chaluka excavation on Umnak Island, ca 3500–4000 YBP [27] and six were from the Mink Island excavation, ca 100–2000 YBP (upper strata) [28] and may or may not be ancestral to the current Alaskan Population.

The 39 OLDRU sea otter samples were obtained from the Kapsyul excavation site on Urup Island in the Kuril Islands of Russia, approximately 100–800 YBP [29]. These are thought to be ancestral to the current sea otter population in the Kuril Islands.

In all bone sampling from pre-fur trade individuals, caution was used to prevent multiple sampling from the same individual and to prevent sample contamination. To minimize the chances of obtaining more than one sample per individual, three precautions were taken: (1) samples were taken from a wide array of sites; (2) a narrow set range of skeletal elements (femur, humerus, mandible, maxilla) was utilized; and (3) after amplification, samples were compared for identical genotypes and, if found, one was removed. Control of potential contamination of the ancestral bone samples followed aspects of protocols described previously [30]–[32]. All materials and equipment that could potentially come into contact with the samples (cotton gauze, tips, tubes, etc.) were treated with UV light for 10 min. Each bone sample was cleaned repeatedly with ethanol, then with 10% bleach and finally rinsed with RNA and DNA free water prior to sampling. A variable speed Dremel™ tool was used, with a new UV-treated drill bit for each sample, to collect bone dust. Samples were collected in sterile 1.5–2.0 mL microcentrifuge tubes and stored at ambient temperature until extraction. Bone samples were decalcified in 1 mL of 0.5 m EDTA for at least 24 h at 37°C. Several changes of EDTA supernatant were made to remove pigmented humic acids absorbed from the sediments. Once relatively clear EDTA supernatant was obtained, the EDTA was removed and the resulting bone pellet was rinsed with sterile water, and the DNA was extracted using the DNeasy tissue extraction kit (Qiagen, Valencia, CA). Multiple efforts were made to authenticate data gathered from otter bone fragments following suggestions made by Paabo et al., 2004 and Gilbert et al., 2005 [33], [34]. These precautions included the following: extraction of DNA and generation of PCR products were in isolation, blank controls were run during DNA extraction and amplification, PCR was run on multiple extracts of the same sample, each sample was run several times (at least in triplicate) to determine accurate scoring of alleles, and finally the alleles generated were within the plausible range of the loci [33], [34].

Microsatellites for bone samples were amplified and screened using a GeneAmp PCR 9600 thermal-cycler (Perkin Elmer, Wellesley, Massachusetts) in 10 µl containing 1 µl of 100–250 ng/µl purified DNA template, 0.5 µM/µl forward and reverse primer, 4 µl PCR Mastermix 2× (Taq polymerase with manufacturer supplied buffer, dNTPs and MgCl_2_), and 4 µl DNA/RNA free dH_2_O to make up final volume. The amplification profile was as follows: one cycle of 94°C (240 s), 35 cycles of 94°C (30 s)+53–57°C (30 s)+72°C (30 s) and one cycle of 72°C (300 s). PCR products were stored at 4°C or −20°C until analysis on an ABI 310 single-capillary system or 3100 sixteen-capillary system in Genescan mode. Allele scoring for each locus was performed using Genotyper Software, version 2.0 or Genescan Software, version 3.0.

Samples from one large geographic area were combined into a presumed intermixing population for statistical testing. For example, all samples from pre-fur trade, Alaska even though taken from different archaeological sites, were combined into one population termed OLDAK.

General descriptive statistics of the loci was determined using GENEPOP 4.0.10 [35]. GENEPOP was used to determine Hardy-Weinberg equilibrium, F statistics (F_IS_ p values and F_ST_ values), heterozygosity, linkage disequilibrium and genotyping failures such as null alleles and other errors. Sequential Bonferroni adjustments were used to determine significance levels for all simultaneous tests making the significance p value 0.01 [36]. MICRO-CHECKER [37] was also used to determine frequency of null alleles and other genotyping errors.

To determine the relative stability of the genetic diversity measured over time BOTTLENECK software was used [38]. This program computes for each population sample, and for each locus, the distribution of the heterozygosity expected from the observed number of alleles under the assumption of mutation-drift equilibrium. The program enables the computation of a P-value for the observed heterozygosity and allele frequency distribution to see whether it is as expected under mutation-drift equilibrium or if there has been a shift provoked by recent bottlenecks [38].

To examine the population structure between geographic regions, we calculated the genetic distance between each region using Nei's standard distance [39] and neighbor-joining methods developed for microsatellite markers, POPULATIONS 1.2.30 [40]. We also employed the program STRUCTURE 2.3.3 [41] to determine distinct populations. This program calculates the likely number of populations (K) and also assigns individuals to populations. Simulation parameters for pre fur trade and modern populations were as follows: 10,000 Burnin period, 2,000,000 MCMC reps after Burnin, and 5 iterations for each K. STRUCTURE is often applied to multiple genetic markers such as microsatellites and can accommodate deviations from Hardy-Weinberg equilibrium such as null alleles. The lowest Ln P(D) or the value closest to zero is the K assumed to be most likely correct [41].

Finally we re-ran GENEPOP 4.0.10 and POPULATIONS 1.2.30 software on geneotypes assigned to populations based on STRUCTURE analysis results to determine potential significant differences in genetic structure between modern and pre-exploitation sea otters. Post STRUCTURE population grouping names were based on the most numerous geographic population of origin. For example a STRUCTURE assigned AK/CA grouping would have primarily AK individuals followed by CA individuals.

## Results

### Genetic diversity

#### Pre-fur trade otters

Departures from Hardy Weinberg expectations were statistically significant for one locus throughout the pre-fur trade populations due to an excess of homozygotes within individuals in all populations, Bonferroni corrected alpha is 0.01 (Table 1). This departure is most likely due to allelic dropout caused by the degraded quality of the pre-fur trade (old) DNA [42]. Both GENEPOP and MICRO-CHECKER estimated no geneotyping errors such as stuttering within any loci but noted null alleles at most loci (average frequency 0.21; range 0.00–0.37). The presence of null alleles within this old DNA is not unexpected, again because of the poor quality of the DNA, and it is not unusual for some alleles to be undetectable.

**Table 1:** Microsatellite statistics of pre-fur trade and modern sea otter populations.

Population	Stat	Mvi57	Mvi87	Mvis72	Mvis75	Lut453	AVE
**OLDCA**	F_IS_ p	0.000	0.020	0.029	0.000	NA	
**N = 98**	He	0.496	0.900	0.833	0.376	0.500	0.621
	A	11	4	3	9	2	5.8
**OLDOR**	FIS p	0.000	0.007	NA	0.000	0.029	
**N = 40**	He	0.775	0.371	NA	0.820	1.000	0.742
	A	9	4	2	12	6	6.6
**OLDWA**	FIS p	0.000	0.471	0.000	NA	0.000	
**N = 34**	He	0.886	0.767	0.794	NA	0.931	0.844
	A	9	5	9	NA	13	9
**OLDAK**	FIS p	0.000	0.000	0.000	0.000	0.000	
**N = 56**	He	0.921	0.872	0.977	0.619	0.929	0.864
	A	15	7	12	4	12	10
**OLDRU**	FIS p	0.000	0.335	NA	0.001	0.162	
**N = 39**	He	0.566	1.000	NA	0.567	0.917	0.762
	A	11	3	1	6	7	5.6
**Total A**		27	14	17	21	20	19.8
**CA**	FIS p	0.018	0.000	1.000	0.174	1.000	
**N = 63**	He	0.624	0.525	0.033	0.774	0.301	0.451
	A	4	4	2	8	2	4
**WA**	FIS p	0.048	0.000	0.346	0.391	1.000	
**N = 33**	He	0.666	0.582	0.401	0.774	0.382	0.561
	A	5	4	2	8	2	4.2
**SEAK**	FIS p	0.152	0.427	0.639	0.680	0.355	
**N = 25**	He	0.736	0.492	0.402	0.753	0.451	0.567
	A	6	3	2	5	3	3.8
**PWS**	FIS p	0.013	0.220	1.000	0.280	0.715	
**N = 35**	He	0.677	0.529	0.399	0.500	0.323	0.485
	A	4	3	2	7	3	3.8
**AM**	FIS p	0.114	0.000	1.000	0.253	0.366	
**N = 40**	He	0.788	0.497	0.281	0.574	0.530	0.534
	A	7	4	2	5	4	4.4
**Total A**		7	6	2	12	4	6.2

**Figure 1:**
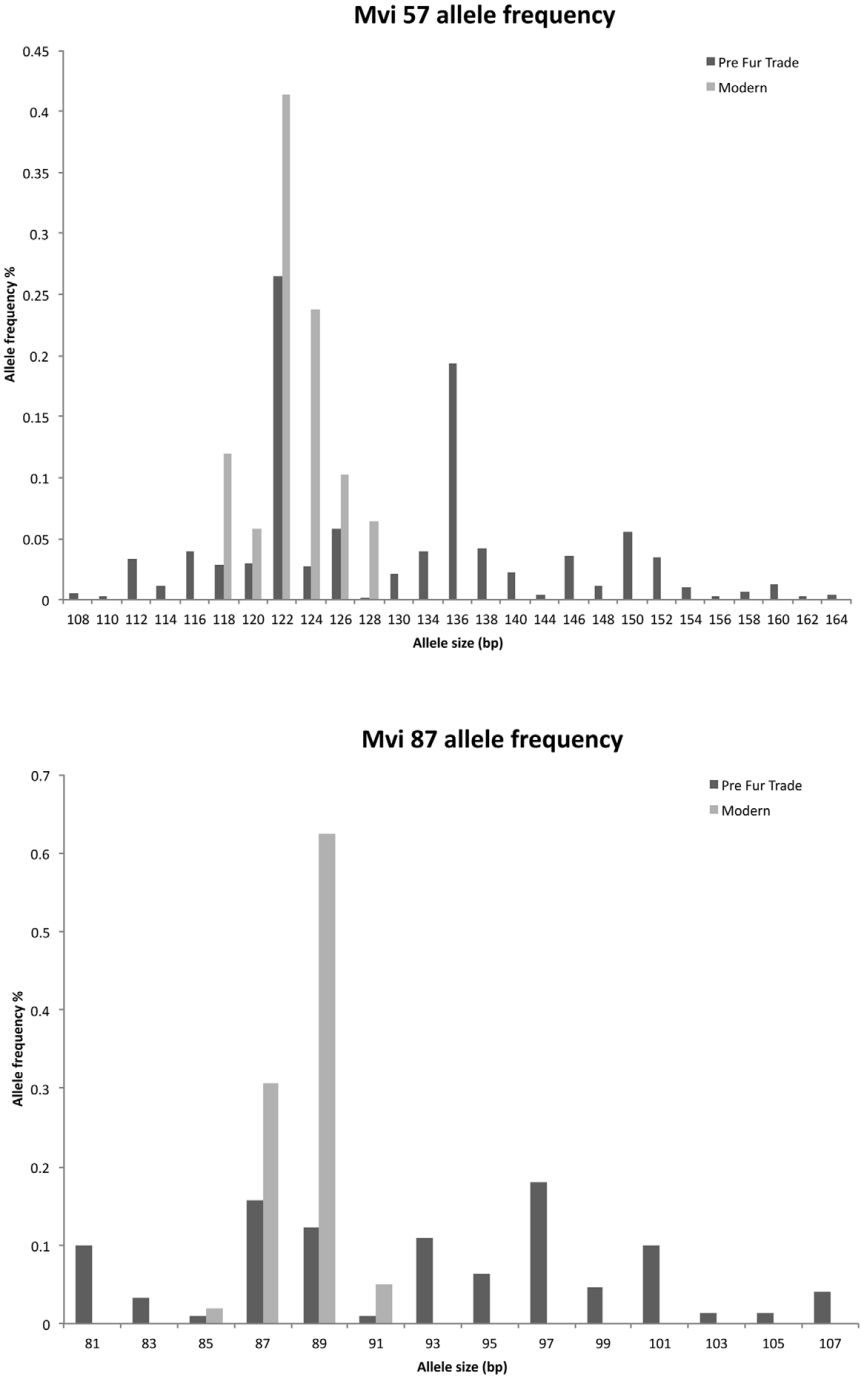
Pre-fur trade and extant sea otter microsatellite allele frequencies in mvi57 and mvi87.

**Figure 2:**
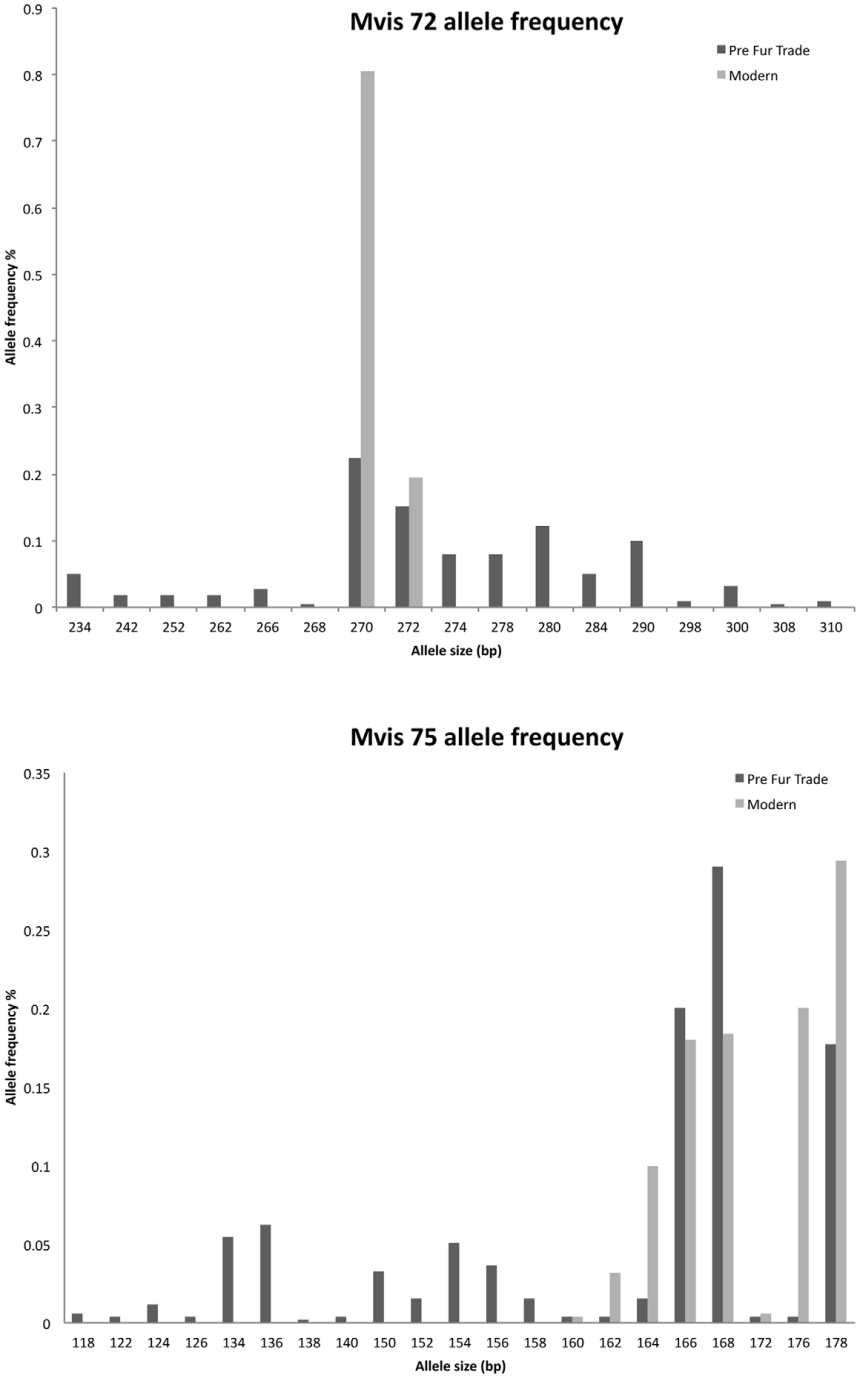
Pre-fur trade and extant sea otter microsatellite allele frequencies in mvis72 and mvis75.

**Figure 3:**
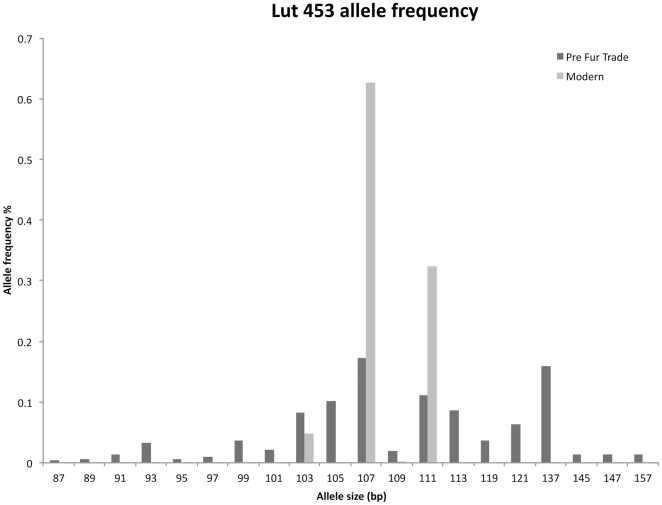
Pre-fur trade and extant sea otter microsatellite allele frequencies in Lut 453.

The average number of microsatellite alleles per locus was 19.80 (range: 14 within Mvi87 to 27 within Mvi57, Table 1, figures 1, 2, 3). The average expected heterozygosity (*H*_E_) was 0.766 (range: 0.621 within OLDCA to 0.864 within OLDAK, Table 1). The total number of alleles observed throughout the five pre-fur trade populations was 89.

#### Modern otters

Departures from Hardy–Weinberg expectations were statistically significant for only 12% of the microsatellite loci throughout all sampled modern otter populations. The only loci out of equilibrium was Mvi87 in CA, WA and AM (excess homozygotes, F_IS_ P = 0.000, Table 1). There were no genotyping failures estimated by GENEPOP and most loci did not have null alleles except for Mvi75 that had an estimated 0.35 null allele frequency.

The average number of microsatellite alleles per locus was 6.2 (range: 2 within Mvis72 and 12 within Mvi75, Table 1, figure 2). Average *H*_E_ was 0.519 (Table 1). The total number of alleles observed throughout the five modern populations was 31 (Table 1). Compared to the pre-fur trade sea otter populations this represents a loss of 33% heterozygosity and 69% of alleles (Table 1 and figures 1, 2, 3).

### Population stability

BOTTLENECK results were statistically significant under the infinite alleles model (IAM) for OLDAK (p = 0.011) and OLDCA (p = 0.016) due to all loci exhibiting heterozygosity deficiency. In contrast, no modern populations were significant for non-expected heterozygote deficiency based on mutation-drift equilibrium under the IAM model.

### Population Structure

Microsatellite *F*_ST_ values were statistically significant between most population comparisons (based on geographic sampling location) regardless of the era (pre-fur trade or modern sea otter populations, Table 2). In samples from the pre-fur trade populations the only non-significant value was the pairwise comparison between OLDCA and OLDRU (Table 2). Within modern sea otters, the only non-significant *F*_ST_ estimates were between populations that were related by translocation such as between the modern founder AM and the related translocated populations SEAK and WA (Table 2). The *F*_ST_ estimates within pre-fur trade otters ranged from a low of 0.031 between OLDRU and OLDCA to a high of 0.274 between OLDWA and OLDCA. Overall the *F*_ST_ estimates among pre-fur trade otters were comparable to those found in modern otter populations (two tailed t-tests assuming unequal variances p = 0.417).

**Table 2:** F_ST_ (below diagonal) and Nei's distance (above diagonal) values for pre-fur trade and modern sea otter populations based on sampled geographic locations and STRUCTURE population assignments.

*Sampled Locations*							
FST pre-fur trade	N	OLDCA	OLDOR	OLDWA	OLDAK	OLDRU	
OLDCA	98	-	1.272	2.068	0.996	0.801	
OLDOR	40	0.133	-	1.238	1.038	1.767	
OLDWA	34	0.274	0.188	-	0.905	2.543	
OLDAK	56	0.225	0.134	0.045	-	1.121	
OLDRU	39	0.031*	0.113	0.203	0.145	-	
FST modern		CA	WA	SEAK	PWS	AM	
CA	63	-	0.251	0.239	0.464	0.271	
WA	33	0.174	-	0.068	0.298	0.095	
SEAK	25	0.170	0.033*	-	0.181	0.044	
PWS	35	0.294	0.185	0.123	-	0.343	
AM	40	0.194	0.061	0.019*	0.215	-	

*STRUCTURE Assignments*							
FST pre-fur trade	N	AK/CA	AK/CA/RU	OR/WA	CA/RU/OR	WA/AK/RU	CA/RU
AK/CA	21/22	-	0.603	4.898	0.654	0.745	1.101
AK/CA/RU	17/16/7	0.015*	-	2.055	0.791	0.661	0.784
OR/WA	19/10	0.273	0.200	-	3.435	1.641	3.721
CA/RU/OR	27/10/5	0.068	0.047	0.337	-	0.914	0.669
WA/AK/RU	18/10/7	0.071	0.016*	0.203	0.143	-	1.134
CA/RU	33/15	0.186	0.148	0.380	0.052	0.267	-
FST modern		CA	PWS/SEAK	AM/SEAK/WA			
CA	66	-	0.464	0.232			
PWS/SEAK	31/10	0.295	-	0.262			
AM/SEAK/WA	33/9/23	0.158	0.165	-			

* = Non-significant *F_ST_* values.

Calculated Nei's genetic distances were significantly higher within pre-fur trade sea otters when compared to values found within modern otters most likely due to the higher number of alleles found within the former (two tailed t-tests assuming unequal variances p<0.001, Table 2).

Population assignment analysis among all individuals for both modern and pre-fur trade otters was constructed using the assignment program STRUCTURE 3.2.2 [41]. Individuals within both pre-fur trade and modern otters were tested for assignment in up to 12 possible populations (K = 1–12), and was run five times for each K to determine consistency (simulation summary for both groups see Table 3). The number of distinct populations that had the highest probability and the lowest Ln P(D) value for pre fur trade otters was K = 6 and for modern otters was K = 3 (Table 3).

**Table 3:** STRUCTURE results* Ln P(D) values for pre-fur trade and modern sea otters.

K value	Pre fur tradeLn P(D)	ModernLn P(D)
1	−1910.62 +/− 0.62	−2120.50 +/− 0.56
2	156 −1569.82 +/− 0.78	−2023.40 +/− 2.30
3	−1388.95 +/− 13.00	**−1894.60 +/− 0.65**
4	−1349.85 +/− 118.73	−1959.48 +/− 13.87
5	−1219.67 +/− 1.44	−1954.10 +/− 18.25
6	**−1178.77 +/− 6.07**	−1976.42 +/− 3.96
7	−1255.90 +/− 37.40	−2050.30 +/− 18.70
8	−1190.78 +/− 4.08	−2121.20 +/−16.10
9	−1260.50 +/− 21.90	−2126.22 +/− 19.92
10	−1308.35 +/− 37.37	−2202.75 +/− 22.68
11	−1236.52 +/− 20.08	−2204.00 +/− 16.50
12	−1200.20 +/− 6.15	−2261.60 +/− 13.65

Bold represents the most likely K based on Ln P(D) value closest to zero (K = 6 for Pre fur trade and K = 3 for Modern).

* Simulation parameters: 10,000 Burnin period, 2,000,000 MCMC reps after Burnin, and 5 iterations for each K.

The STRUCTURE assigned populations were analyzed for population differences and gene flow as a comparison with the geographically assigned groups. The STRUCTURE populations were named based on the most abundant geographic locations that made up at least 75% of the group. For example the STRUCTURE assigned AK/CA had primarily AK individuals followed by CA individuals, while the AK/CA/RU group had primarily AK, followed by CA, then followed by RU in abundance (Table 2). The STRUCTURE assigned total population sample sizes (N) were slightly smaller than the geographically assigned samples sizes because of the few assigned individuals that did not belong to the major groups that were represented in the name were not included for further analysis for population differentiation.

For population structure based on STRUCTURE assignments, microsatellite *F*_ST_ values were statistically significant between most population comparisons regardless of the era (pre-fur trade or modern sea otter populations (Table 2). In pre-fur trade populations the only non-significant values were the pairwise comparisons between AK/CA and AK/CA/RU (*F*_ST_ = 0.015) and WA/AK/RU and AK/CA/RU (*F*_ST_ = 0.016, Table 2). Overall the *F*_ST_ estimates among pre-fur trade otters and modern otters were not statistically different between groups based on geographic sampling location or based on STRUCTURE assignments (*Fst F* = 0.072, p = 0.791 and distance *F* = 0.218 p = 0.644) and for modern (*Fst* F = 1.106, p = 0.315 and distance F = 1.195 p = 0.297).

Finally the *Fst* values were not significantly different when the remnant groups were compared using sampled or structure assigned populations (*F* = 1.151, p = 0.344).

## Discussion

The greatest impact of the fur trade of the 18th and 19th centuries on sea otter genetics is the dramatic loss of genetic diversity. Modern populations have lost almost half their genetic heterozygosity and over 66% of the number of alleles within microsatellite loci. The numbers of alleles found within pre-fur trade samples was significantly larger than that found within extant populations. In addition the number of alleles found in the pre fur trade populations are likely an underestimate of the true number due to the null alleles found in ancient samples. Their presence underestimates genetic diversity, which generally results in increased *F_ST_* and genetic distances values [42]. However since there is a large difference in numbers of alleles between the ancient and extant populations, even if it there is an underestimate in the pre fur trade population, we believe these datasets are acceptable for the comparison of genetic variation and population differences. Thus the *F_ST_* estimates and genetic distances found among pre fur trade otters are likely inflated due to the effect of null alleles and should be regarded as conservative when interpreting population structure and gene flow.

*F_ST_* estimates among pre-fur trade and modern sea otters were comparable and not statistically significantly different. Populations that are directly comparable between ancestral and modern groups are the OLDCA and OLDAK populations and the current remnant populations CA and the remnant groups from Alaska (PWS and AM). For example the *F_ST_* estimate between OLDCA and OLDAK was 0.225 while the *F_ST_* estimate between CA and PWS was 0.294 and between CA and AM was 0.194 (Table 2). Even though modern populations have lost significant genetic diversity it seems that they have retained population structure after the fur trade extirpations.

Most *F_ST_* estimates between most ancestral or modern groups are equivalent or less than 0.20 (Table 2). Avise 1994 [43] stated that an *F*_ST_ of 0.20 corresponds to an average exchange of one individual per generation, and that *F*_ST_ estimates lower than 0.20 are described as “high gene flow species”. This result is expected if ancestral sea otter populations were relatively uninterrupted along their historic range. However, it is surprising that modern *F*_ST_ numbers are still relatively low, even though these populations are thought to have been geographically isolated for more than over 100 years. The greatest geographic distance prohibiting the migration of sea otters between extant groups is the 1400 km distance between California and Washington, much farther than sea otters typically migrate [8]. However, the extant Northern populations are separated, for the most part, by geographic distances that sea otters are capable of crossing. For example, the Washington and the Vancouver Island, BC population is separated by only 120 km while sea otters have been documented migrating over 400 km [8]. In addition, groups of sea otters from Southeast Alaska to Central Alaska and to the Aleutian chain are separated by distances that sea otters are capable of migrating and may be thought of as relatively contiguous populations [7], [8]. The relatively small distances between sea otter populations in the northern parts of the range in conjunction with the genetic similarities found between founder and translocated groups, such as between AM and SEAK or WA, may account for the relatively low *F*_ST_ values found within the modern sea otters sampled here.

Overall, genetic tests performed on population structure using all methods reported here are similar between pre-fur trade and modern sea otters. Again this is surprising given the sea otters history of extirpation and population fragmentation. Even though sea otters lost over 99% of their numbers due to fur trade exploitation they have retained much of their historical genetic structure. The main difference between pre-fur trade and modern populations, aside from genetic diversity, is that some *F_ST_* and genetic distances are larger among the pre-fur trade populations, particularly when OLDOR and OLDWA are compared to other OLDCA and OLDRU (Table 2). We believe this is most likely due to the higher diversity in pre-fur trade samples enabling greater differentiation between groups (Table 1, figures 1, 2, 3). In addition the allelic frequency differences and distribution between the pre-fur trade populations in the center of the range (OLDOR and OLDWA) when compared to the ends of the range (OLDCA, OLDRU and any geneotypic combinations made by STRUCTURE that include AK with CA and/or RU) may be the result of clinal variation. Allelic frequencies may differ in the center of the range when compared to the ends of the cline, due to the gradual and continuous change of allelic frequencies over the large geographic area that sea otters were sampled. We did not sample all contiguous pre-fur trade populations and easily may have missed documenting the gradual continuous change in alleleic frequencies between populations at the ends of the cline and the middle.

We did document some gene flow between the end of the geographic range of the sea otter and the center. For example, STUCTURE analyses assigned OLDOR samples to both OLDCA and OLDWA. However the majority of OLDOR were assigned to the group containing OLDWA samples suggesting more gene flow moving northwards rather than in a southerly direction (Table 2). These results suggest that using nuclear markers employed here, the OLDOR may have experienced more gene flow from Northern groups and thus be more similar to the OLDWA population rather than OLDCA. This finding using nuclear markers is in contrast to the finding made by Valentine et al.,2008 [44] who used mtDNA from ancient OR samples. They documented more matches with the typical CA mtDNA haplotype rather that those typically found in Northern sea otters, *E.l. kenyoni*, and concluded that the ancient OR sea otters were likely the Southern sea otter subspecies, *E.l. nereis* [44]. However they did document some samples with the typical Northern sea otter haplotype suggesting geneflow both to the north as well as to the south. Perhaps females from the ancient OR population were derived primarily from southern animals while the males mating with them may have migrated primarily from the north. This would explain the assignment of many OLDOR to OLDWA in this study using nuclear markers and why Valentine et al. 2008 [44] was unable to detect the male driven geneflow from the north because of the maternal inheritance quality of mtDNA. It is unknown if or where there was a hybrid zone between southern and Northern sea otters in Oregon. More work needs to be done with finer scale sampling along the Oregon coast to determine if there indeed was a significant hybrid zone between northern and southern sea otters.

Although modern sea otters retain less than half the genetic diversity they once had, the populations with the greatest diversity today are the translocated populations founded by a mix of two populations (SEAK founded by both AM and PWS) [6], [7]. The CA population is unique in that it historically and currently has the lowest genetic diversity, indicating bottlenecks predating the fur trade as suggested by Aguilar et al. 2008 [16]. BOTTLENECK analyses supported this hypothesis in both OLDCA and OLDAK. These pre-fur trade bottlenecks may have been caused by extirpations due to extensive harvesting by local people.

The modern translocated groups that are founded by two populations are the populations with the highest growth rates and the largest, healthiest otters. Perhaps one way to assist the threatened populations with the lowest diversity such as CA would be to boost their genetic diversity by encouraging historical gene flow through future translocations. The future health of sea otter populations is not certain under current conditions but the maintenance and enhancement of the remaining genetic diversity is crucial and should be a high priority in any management plan.
